# Prevalence of pain flashbacks in posttraumatic stress disorder arising from exposure to multiple traumas or childhood traumatization

**DOI:** 10.1080/24740527.2018.1435994

**Published:** 2018-02-21

**Authors:** B. Macdonald, T. V. Salomons, L. Meteyard, M. G. Whalley

**Affiliations:** a Department of Psychology and Clinical Language Sciences, University of Reading, Reading, UK; b Berkshire Traumatic Stress Service, Berkshire Healthcare NHS Foundation Trust, Reading, UK

**Keywords:** pain, flashbacks, PTSD, somatosensory, multisensory

## Abstract

**Background:**

Flashbacks are a form of multisensory memory that are experienced with a “happening in the present” quality. Pain flashbacks are a re-experiencing of pain felt at the time of a traumatic event. It is unclear how common pain flashbacks are.

**Aims:**

The current study was designed primarily to assess the prevalence of pain flashbacks in a sample of patients with posttraumatic stress disorder (PTSD).

**Methods:**

We assessed the prevalence of pain flashbacks over a period of 2 years in patients (*n* = 166) referred to a psychological trauma service in the UK. Patients underwent a clinical screen for PTSD and completed a self-report measure of pain flashbacks.

**Results:**

Pain flashbacks were classified as present in 49% of a sample of complex trauma patients meeting criteria for PTSD. Pain flashbacks were positively associated with the extent of pain at the time of trauma.

**Conclusions:**

Pain re-experiencing in PTSD, and its relative absence in nonclinical populations, supports an account of memory in which perceptual details can be re-experienced when memories have been encoded under conditions of extreme stress. It may be possible to conceptualize some cases of unexplained pain as pain flashbacks or of having a trauma origin.

## RÉSUMÉ

**Contexte**: Les flashbacks sont une forme de souvenir multisensoriel qui se manifeste en donnant l’impression de se dérouler dans le présent. Les flashbacks douleureux sont une reviviscence de la douleur ressentie au moment d’un évènement traumatique. On ne sait pas à quel point les flashbacks douleureux sont communs.

**But**: La présente étude a été conçue principalement pour évaluer la prévalence des flashbacks douleureux au sein d’un échantillons de patients souffrant d’un trouble de stress post-traumatique (TSPT).

**Méthodes**: Nous avons évalué la prévalence des flashbacks douleureux sur une période de deux ans chez des patients (n = 166) référés à un service spécialisé dans les traumstismes psychologiques en Grande-Bretagne. Les patients ont été soumis à un dépistage du TSPT et ont répondu à un questionnaire portant sur les flashbacks douleureux.

**Résultats**: Les flashbacks douleureux étaient présents chez 49 % des participants d’un échantillon complexe de patients souffrant de traumatismes qui répondaient aux critères pour le TSPT. Une association positive a été observée entre les flashbacks douleureux et le degré de douleur ressenti au moment du traumatisme.

**Conclusions**: La reviviscence de la douleur dans les cas de TSPT et son absence relative au sein de populations non cliniques semblent démontrer que les détails perceptuels d’un souvenir peuvent être revécus lorsqu’ils ont été encodés dans des conditions de stress extrême. Certains cas de douleur inexpliquée peuvent être considérés comme des flashbacks douleureux, ou comme ayant une origine traumatique.

## Introduction

Flashbacks are a distinguishing symptom of posttraumatic stress disorder (PTSD)^1,2^ in which intrusive memories of a past traumatic event are involuntarily re-experienced.^3,4^ They have a temporal component whereby they are re-experienced as if they were happening in the present (*nowness*), and the individual may behave as if the event were occurring in that moment.^3,5^ Flashbacks are described by PTSD patients as being brief, perceptually detailed, and containing more sensory details than nontrauma memories.^1,6–11^ Flashbacks in PTSD have been distinguished from intrusive memories in other conditions by characteristics of nowness, a less coherent narrative structure, and their containing more sensory details.^12^ Neurologically, flashbacks in PTSD have been associated with alterations in brain structure and function.^4,13^

Case reports have described the presence of pain flashbacks in patients with PTSD. Pain flashbacks are forms of somatosensory memory in which pain experienced at the time of a trauma is re-experienced when triggered later, despite the apparent absence of persisting injury. Case examples have included a soldier who lost his eye and who reported re-experiencing the pain he had felt during his time in hospital,^14^ two patients who regained consciousness during surgical anesthesia and who later re-experienced pain felt during their operations,^15^ and a survivor of the 2005 London bombings who, when reminded of the trauma, reported re-experiencing a burning pain in his arms first felt during the explosion.^16^ Somatosensory re-experiencing of pain appears to be unique to PTSD. For example, no participants in a large study examining pain memory in a nonclinical sample reported any sensory re-experiencing of pain despite frequently being able to retrieve declarative memories for painful events,^17^ a finding that has subsequently received further support.^18^

PTSD is often comorbid with physical problems such as chronic pain and shares common symptoms including hypervigilance, avoidance behaviors, and elevated somatic focus.^19,20^ It has been suggested that PTSD–pain comorbidity may be due to a shared vulnerability,^19^ mutual maintenance,^21^ or a combination of mechanisms.^19^ In the case of pain resulting from a trauma that gave rise to PTSD, the mutual maintenance model suggests that pain serves as a reminder of the trauma, potentially triggering flashbacks. These may be experienced as painful, subsequently increasing associated attentional, cognitive, physiological, and behavioral responses (i.e., increased attention to pain stimuli, hyperarousal, avoidance), potentially entering into a self-amplifying cycle. In addition, the shared vulnerability model suggests that chronic pain and PTSD are associated with shared predispositional factors, increasing the likelihood of comorbidity. If pain is experienced as part of a flashback in PTSD, it is possible that flashback duration and intensity are increased through factors included in the mutual maintenance model. In addition, if patients are also suffering from chronic pain, flashback frequency (including pain flashbacks) may also be increased.

Pain flashbacks (somatic flashbacks) have the potential to explain *some* cases of chronic, unusual, or medically unexplained pain.^19,22^ For example, links have been made between trauma history and certain subtypes of pelvic pain,^23^ and high rates of trauma history have been observed in pelvic pain patients.^24^ Researchers have hypothesized that some cases of phantom limb pain might be conceptually similar to the trauma memories described above,^25^ and memory-focused techniques have reportedly been used successfully to treat such pains.^26^ Awareness that patients with PTSD may re-experience unusual body sensations means that these can be openly discussed, formulated within a trauma model, and then appropriately targeted with psychological treatments.

It is not currently known how prevalent pain flashbacks are or what factors are associated with their occurrence. The current study was designed to investigate the prevalence of pain flashbacks in a population of patients diagnosed with PTSD.

## Materials and methods

### Participants

The present research consists of an analysis of data collected in routine clinical practice and was approved by a clinical audit team as a service evaluation not requiring patient consent (Berkshire Healthcare NHS Foundation Trust project reference number 2738). Data were collected in a tertiary psychological trauma service in the UK that specializes in the treatment of complex trauma (simpler cases of trauma in the locality are treated in a primary care service). Data from all patients referred to the service between January 1, 2013, and January 1, 2015, were eligible for inclusion in the study. Data for the study consisted of information collected from an initial assessment appointment, the purpose of which was to determine whether patients had PTSD and whether they were suitable for treatment in the specialist trauma service.

### Procedure

Patients attended a one-hour psychological assessment conducted by a qualified clinician specializing in the treatment of trauma. All clinicians held a doctorate in clinical psychology. The purpose of the appointment was to assess whether patients met diagnostic criteria for current PTSD according to *Diagnostic and Statistical Manual of Mental Disorders*, fourth edition (DSM-IV) criteria^27^ and whether they were immediately suitable for treatment in the trauma service or would be best served by a referral to another service. Semistructured interviews were used to assess whether patients had been exposed to single or multiple experiences of trauma, to assess whether they had experienced sexual trauma, to assess current symptoms, and to determine therapeutic goals. Because the study was an evaluation of data collected in the course of standard clinical practice, measures of interrater reliability to check diagnostic congruence were not collected. Psychometric measures were routinely administered to all patients. These were sent to patients in advance of the assessment appointment. If patients did not complete them prior to the appointment, they were invited to complete them in the waiting room on the day of the appointment.

### Questionnaires and measures

#### The Posttraumatic Diagnostic Scale

The Posttraumatic Diagnostic Scale (PDS)^28^ is a widely used self-report measure of PTSD. Items measuring each of the 17 PTSD symptoms (according to DSM-IV criteria) are rated for the past month on a 0–3 scale. The PDS has demonstrated satisfactory test–retest reliability, internal consistency, and convergent and concurrent validity.^28^

#### The Hospital Anxiety and Depression Scale

The Hospital Anxiety and Depression Scale (HADS)^29^ is a widely used self-report measure of anxiety and depression. Fourteen items measuring depression and anxiety symptoms are rated for the past week on a 0–3 scale. The HADS has demonstrated satisfactory psychometric properties in terms of factor structure, intercorrelation, homogeneity, and internal consistency^30^ and has been found to perform well in assessing symptom severity and caseness of anxiety and depression,^31^ although use of the HADS as a reliable measure of depression has also been questioned by some authors.^32^

#### Pain flashbacks measure

**Figure 1.**
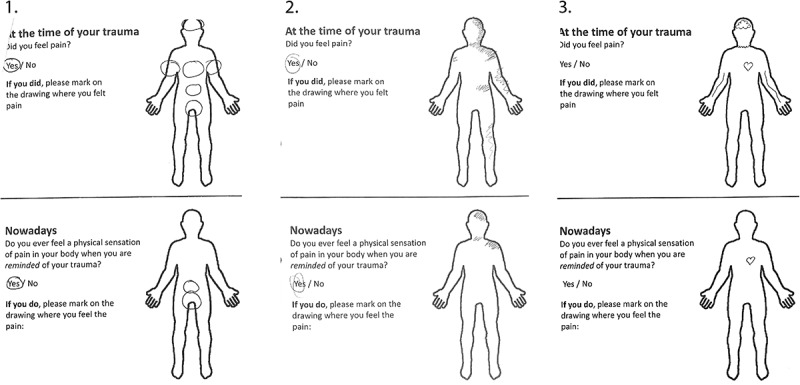
Completed examples of the pain flashbacks measure. Panels 1 and 2 represent relatively clear delineation of pain overlap and both scored positively for pain flashbacks. Panel 3 represents an example of failure to answer the yes/no questions and possible presence of emotional pain.

A one-page measure was developed that explored whether patients had experienced pain at the time of their trauma (yes/no response) and whether they experienced pain when reminded of their trauma (yes/no response). If participants marked yes to either of these questions, they were asked to mark on a corresponding body outline the location where they experienced this pain (see Figure 1).

### Data analysis

#### Pain flashbacks criteria

Three of the authors met to determine the criteria by which pain flashbacks should be judged to be present (B.M., T.S., M.W.). Pain flashbacks were judged to be present if

- a patient indicated that he or she experienced pain at the time of trauma;
- a patient marked yes to the question asking whether he or she experienced pain upon reminders; and
- the authors judged that there was substantial overlap between the reported locations of pain experienced at the time of trauma and pain experienced upon reminders, as drawn by patients on a body outline. For example, a patient reporting pain in the genital region at the time of trauma and upon remembering was counted as overlap. If multiple body regions were marked, overlap had to be present in at least one region. Overlap was judged by eye and required a consensus agreement among the authors to be judged as present.

Pain flashbacks were judged not to have been present if a patient clearly disambiguated his or her report as an emotional reaction (e.g., by drawing a broken heart or referring to emotional pain using text). Examples of completed pain flashback measures are given in Figure 1.

#### Pain location coding

Location of reported pain was coded for the two time points (pain at time of trauma, pain upon remembering). In order to parsimoniously code the spatial extent of pain experienced during trauma, the body was split into five locations: head, peripheries (arms, legs), torso, genitals, and heart. The genital region was included because of the prevalence of sexual abuse in the sample. The heart region was included as separate from the torso to evaluate whether patients were attempting to indicate the presence of emotional pain. These locations were not mutually exclusive, so we then summed across the regions to obtain an ordinal measure of the spatial extent of pain at the time of trauma. That is, individuals with pain in one region would have a score of 1, and individuals with pain in all regions would have a score of 5.

#### Statistical analysis

##### Prevalence of pain flashbacks

In total, between January 1, 2013, and January 1, 2015, 610 patients were referred to the specialist trauma service; 465 were suitable referrals and were offered an assessment appointment. Assessment appointments were not attended by 141 of patients, 71 were assessed but not accepted for treatment (typically either because they did not have PTSD or because PTSD was not their primary presenting problem), and 253 were accepted for treatment within the service (i.e., they were diagnosed with PTSD and were suitable for treatment). A final sample of 166 attended the assessment, were accepted for treatment, and completed the pain flashbacks measure (87 did not complete the pain flashbacks measure). Demographics for both groups are provided in Table 1. To evaluate the prevalence of pain flashbacks, we counted the number of participants who met criteria for the presence of pain flashbacks (see above) in the group that completed the pain flashbacks questionnaire (*n* = 166).

**Table 1.** Demographic data of participants with PTSD who completed the pain flashbacks measure. For comparision, the 87 who did not complete the measure are also presented.

Variable	Completed the pain flashbacks measure	Did not complete the pain flashbacks measure	Effect size^a^
*N*	166	87	—
Age	37.06 (SD = 14.43; range = 18–68)	40.09 (SD = 11.66; range = 18–68)	0.23
Sex	46 M (28%)/120 F (72%)	21 M (24%)/66 F (76%)	0.13
Index trauma	CSA 57 (34%), DV 29 (17%), medical 13 (8%), military 2 (1%), rape 13 (8%), RTA 4 (2%), sexual assault 3 (2%), torture 5 (3%), traumatic bereavement 4 (12%), violence 20 (12%), other 16 (10%)	CSA 32 (37%), DV 11 (13%), medical 8 (9%), military 4 (5%), rape 6 (7%), RTA 3 (3%), sexual assault 1 (1%), torture 1 (1%), traumatic bereavement 5 (6%), violence 5 (6%), other 11 (13%)	—
Single incident vs. multiple trauma	20 single (12%), 146 multiple trauma (88%)	15 single (17%), 72 multiple trauma (83%)	0.05
PDS	37.26 (SD = 7.79)	36.95 (SD = 8.59)	0.04
HADS (Anxiety)	15.43 (SD = 3.60)	15.4 (SD = 3.63)	0.01
HADS (Depression)	11.24 (SD = 4.45)	11.27 (SD = 4.61)	0.01

^a^Calculated as Cohen’s *d* for continuous variables and phi (φ) for chi-square.

CSA = childhood sexual abuse; DV = domestic violence; RTA = road traffic accident; PDS = Posttraumatic Diagnostic Scale; HADS = Hospital Anxiety and Depression Scale.

##### Factors associated with the presence of pain flashbacks

We tested for relationships between each psychometric assessment (PDS total score, HADS Anxiety, HADS Depression) or trauma characteristic (whether the trauma was sexual in nature, whether the trauma occurred during childhood, whether PTSD was from a single trauma or multiple traumas, the variable coding the spatial extent of trauma pain) and the presence of pain flashbacks (yes or no). To compare values for the two groups (pain flashbacks vs. no pain flashbacks), we used *t* tests for normal continuous variables and the Wilcoxon rank sum test for ordinal and nonnormally distributed continuous variables. We used chi-square for binary variables.

## Results

### Prevalence of reported pain and pain flashbacks

The proportion of patients who reported experiencing pain at the time of trauma, the proportion reporting experiencing pain upon remembering, and the proportion meeting criteria for pain flashbacks are given in Table 2. Of the total sample (*n* = 166), 82 met the criteria for pain flashbacks. The locations of reported pain for different time points and groups are given in Table 3 along with trauma characteristics and psychometric data.

**Table 2.** Proportion of the total sample reporting pain flashbacks.

Description	Number	Percentage of total sample
Total sample (met PTSD criteria and completed pain flashback measure	166	100
*(of these)*		
Reported pain at time of trauma (*t*1)	124	74
*(of these)*		
Reported pain upon remembering (*t*2)	91	55
*(of these)*		
Overlap of pain locations at *t*1 and *t*2	82	49

PTSD = posttraumatic stress disorder.

**Table 3.** Spatial extent of pain, trauma characteristics, and psychometric data for patient subgroupings. Reported pain locations not mutually exclusive—patients could report pain at more than one location.

Participants who reportedor met criteria for	*N*	Number reportingpain at location	Trauma characteristics	Psychometric data
Pain at time of trauma	124	Head = 89 (72%) Torso = 73 (59%) Periphery/limb = 71 (57%) Genitals = 62 (50%) Heart = 28 (23%)		
Pain upon remembering	109	Head = 62 (57%) Torso = 61 (56%) Periphery/limb = 36 (33%) Genitals = 38 (35%) Heart = 26 (24%)		
Pain flashbacks	82	Head = 62 (76%) Torso = 52 (63%) Periphery/limb = 45 (55%) Genitals = 41 (50%) Heart = 16 (20%)	Childhood trauma = 61 (74%) Sexual trauma = 49 (60%) Single-event trauma = 6 (7%) Multiple trauma = 76 (93%)	PDS = 37.49 (SD = 7.36) HADS Anxiety = 15.33 (SD = 3.32) HADS Depression = 11.07 (SD = 4.14)
No pain flashbacks	84	Head = 42 (50%) Torso = 31 (37%) Periphery/limb = 9 (11%) Genitals = 27 (32%) Heart = 17 (20%)	Childhood trauma = 56 (67%) Sexual trauma = 55 (65%) Single-event trauma = 14 (17%) Multiple trauma = 70 (83%)	PDS = 37.01 (SD = 8.29) HADS Anxiety = 15.55 (SD = 3.89) HADS Depression = 11.43 (SD = 4.77)

PDS = Posttraumatic Diagnostic Scale; HADS = Hospital Anxiety and Depression Scale.

### Factors influencing flashback pain

The group that did not complete the pain flashbacks measure (*N* = 87) and the group that did (*N* = 166) did not differ in the distribution of gender (χ^2^ = 4.10, *P* > 0.05), single incident or multiple trauma (χ^2^ = = 0.89, *P* > 0.05), or mean scores for the PDS, *t*(114) = −0.35, *P *> 0.05, HADS Anxiety, *t*(119) = −0.09, *P *> 0.05, or HADS Depression, *t*(116) = 0.003, *P *> 0.05. The groups did differ significantly by age at assessment, *t*(181) = 2.04, *P *< 0.05, with the group who completed the pain flashbacks questionnaire being 3 years older on average (see Table 1). Therefore, the group that completed the pain flashbacks questionnaire is representative of the typical clinical case load over the 2-year period and did not differ meaningfully from the group that did not complete the questionnaire (aside from being approximately 3 years older).

We explored whether there were relationships between the presence of pain flashbacks and the measures from psychometric assessments and trauma characteristics. Not all participants had background data for all variables; therefore, sample sizes differed slightly across comparisons. Sample sizes are provided in Table 4. Pain flashbacks are coded as a binary variable, and psychometric assessments are continuous (PDS total score, HADS Anxiety, HADS Depression; see below). Normality tests showed that PDS total and HADS Anxiety were not normally distributed (respectively Shapiro-Wilk = 0.96 and 0.95, both *P *< 0.05) and HADS Depression was normally distributed (Shapiro-Wilk = 0.98, *P *> 0.05). Characteristics of trauma variables are binary or ordinal. These are whether the trauma was sexual in nature (binary, sexual vs. nonsexual), whether the trauma occurred during childhood (binary, child vs. adult), whether PTSD was from a single trauma or multiple traumas (binary, single vs. multiple), and the variable coding the spatial extent of trauma pain (ordinal, number of locations coded for pain, number between 0 and 5). Results from this analysis are presented in Table 4, including reporting of test statistics and effect sizes. Spatial extent of trauma pain was significantly different between the pain flashbacks and no pain flashback groups, *W*(148.47) = 2351, *P *< 0.001, with more locations of trauma pain in the former (pain flashbacks group = 2.63 [1.17]) than the latter (no pain flashbacks group = 1.86 [1.68]). Single or multiple trauma showed a small effect that did not reach significance (χ^2^ = 2.59, *P* = 0.1), with fewer people in the pain flashbacks group having single traumas (6 vs. 74) compared to the no pain flashbacks group (14 vs. 70).

**Table 4.** Testing relationships between predictor and outcome variables.

Variable	Pain flashbacks group	No pain flashbacks group	Test statistic	Effect size^a^
PTSD severity	M = 37.49 (7.35), *n* = 72	M = 37.01 (8.28), *n* = 67	*W* (132.21) = 2301, *P* = 0.64	0.061
Anxiety	M = 15.33 (3.32), *n* = 73	M = 15.55 (3.89), *n* = 73	*W* (140.49) = 2755, *P* = 0.72	−0.061
Depression	M = 11.06 (4.14), *n* = 73	M = 11.43 (4.76), *n* = 72	*t*(139.7) = 0.49, *P* = 0.63	−0.082
Childhood trauma	Yes = 61	Yes = 56	χ^2^(1) = 0.85,	0.071
	No = 21	No = 28	*P* = 0.35	
Sexual trauma	Yes = 49	Yes = 55	χ^2^(1) = 0.36,	0.047
	No = 33	No = 29	*P* = 0.55	
Single trauma	Yes = 6	Yes = 14	χ^2^(1) = 2.59,	0.125
	No = 74	No = 70	*P* = 0.1	
Extent of trauma pain	M = 2.63 (1.17), *n* = 84	M = 1.86 (1.68), *n* = 82	*W* (148.47) = 2351, *P* = 0.0003*	0.53

^a^Calculated as Cohen’s *d* for continuous variables and phi (φ) for chi-square.

*P < 0.001.

## Discussion

Pain at the time of trauma was reported by 74% of the present sample of clients with PTSD, and 74% of these reported experiencing pain when reminded of their trauma. Pain flashbacks were classified as present in 49% of the present sample. The proportion of patients reporting pain flashbacks is consistent with previous research documenting the relatively frequent occurrence of somatosensory re-experiencing in PTSD.^8^ Analyses indicated that the spatial extent of pain experienced during the trauma was positively associated with a report of pain flashbacks.

There are two important implications from our finding indicating a high proportion of pain flashbacks in a sample of patients with PTSD. The first is that individuals with PTSD appear to be capable of experiencing pain through a mechanism likely to involve memory rather than nociceptive pathways. Given the comorbidity between PTSD and chronic pain, it is possible that *some* patients with symptoms of refractory chronic pain may be experiencing pain flashbacks. A similar idea has been advanced by other authors, with a suggestion that headaches and other pains in patients with PTSD may function as somatic flashbacks.^19,22^ If the present finding can be replicated, it will be important for pain clinicians to accurately identify pain flashbacks in patients with chronic pain. Given concerns about the rising use of opiates in the treatment of chronic pain,^33^ we conjecture that evidence-based pain management may prove ineffective for flashback pain, whereas there are indications that psychological treatments that help other posttraumatic memory symptoms are likely to be beneficial.^26,34^

The second implication concerns models describing memory re-experiencing in PTSD. One class of theories has argued that recurrent involuntary memories occur when autobiographical content has become particularly accessible in memory. According to this account, PTSD flashbacks are seen to be the result of normal memory mechanisms: Normal memories can be both voluntarily and involuntarily recalled, and PTSD memories are just particularly available to both voluntary and involuntary recall.^35,36^ An opposing view draws attention to particular characteristics of flashbacks in PTSD, including the sense of nowness and the more prominent sensory qualities.^1,11^ Proponents of this view argue that ordinary memory mechanisms operate differently under conditions of extreme stress, resulting in the characteristics of memory observed in PTSD. One such position is taken by dual representation theories of memory in PTSD^12^ that discriminate between two forms of memory storage: contextual (which is to some extent dependent on hippocampal function) and sensory (which is less hippocampally dependent). Under normal circumstances, both forms of memory are encoded and the contextual memory inhibits the sensory component, leading to events being remembered but not re-experienced. During a traumatic event, encoding of contextual information is said to be impaired, which, in PTSD, leads to uninhibited sensory components and memories that are re-experienced rather than just remembered. In the case of pain flashbacks, the dual representation theory would argue that the strong sensory (pain) representation has been encoded and is not being sufficiently inhibited by a strong contextual representation. The prevalence of pain re-experiencing in patients with PTSD and its apparent absence in nonclinical populations^17,18^ supports an account of memory in PTSD in which sensory properties are especially prominent.

### Limitations and recommendations

The present study was an evaluation of data collected in routine clinical practice, and we believe that our report of pain flashback prevalence is likely an overestimate of the true rate in the population of individuals with PTSD. First, we selected our sample from a trauma service specializing in complex trauma rather than a true community sample. Our sample had a higher proportion of multiple and childhood trauma. Secondly, we did not assess for the presence of other pain conditions, which may have biased reports of current pain. The possible presence of continued injury (e.g., neuropathy from original trauma), widespread pain disorder (e.g., fibromyalgia), or constant pain (i.e., experience of pain upon remembering but also at other times), all of which might be exacerbated by PTSD, could have contributed to elevated reports of pain in the present and/or upon remembering. It is also possible that some pain reports, at both time of trauma and time of remembering, represent generic stress-related pain unrelated to either traumatic injury or its re-experiencing (e.g., headaches, tightening of the chest). Finally, stress-related factors are known to elevate current pain report, necessitating an accurate assessment of current pain conditions. Nevertheless, it is unlikely that these factors alone account for the high prevalence of pain re-experiencing reported here, suggesting that pain flashbacks are more common than previously considered.

Other limitations of this study included its use of a novel measure that assessed pain flashbacks indirectly. Our measure did not measure the intensity or fear of pain at time of trauma or upon remembering or the participants’ judgments about the subjective similarity of the pain at the two time points. The absence of these factors prevented us from knowing with certainty whether each patient experienced a true pain flashback. In order to improve the accuracy of future pain flashback assessments, we would make a number of recommendations.

### Changes to screening

We recommend that future studies of pain flashbacks include an assessment of pre-existing pain conditions, coexisting pain conditions, and the patient’s peritraumatic experience including dissociation and feelings of helplessness. We would predict that peritraumatic dissociation would influence the formation of later flashbacks and that only pain that patients are helpless to do anything about would predict pain flashbacks.

### Changes to the pain flashback measure

An improved pain flashback measure would ask patients to shade (instead of mark) areas on body outlines to represent pain, allowing more accurate calculation of the potential overlap between pains at the time of the trauma and upon remembering. Front and back body images for each time point (pain at trauma, pain upon remembering) would enable more accurate pain assessment, as would ratings of pain intensity at the time of trauma for each pain location (to allow analysis of whether only the most severe pains go on to become pain flashbacks). Obtaining more detailed descriptors of the pain (e.g., sharp, dull) would allow for more refined judgment about whether a particular pain sensation was being re-experienced, as would asking patients to rate the similarity of pain flashbacks to pain experienced at time of trauma (e.g., see Katz and Melzack^25^). In the present study, it was not clear whether some pains (e.g., head or heart) represented true physical pain, feelings like stress-induced headaches, or emotional pain. It would be helpful to refine the measure to distinguish these.

### Evaluation of psychometric properties of the measure

As part of a formal evaluation of the psychometric properties of an improved measure of pain flashbacks, we would recommend the inclusion of a clinician interview as part of the assessment. This would allow for the inclusion of behavioral measures or a provocation task to complement self-report.

### Summary

To our knowledge, the present study is the first attempt to assess the prevalence of pain flashbacks in patients with PTSD. In our sample of complex trauma patients, pain flashbacks were classified as present in 49% of the sample, and although this likely represents an overestimate of the prevalence in a community sample of PTSD, it acts as an upper bound. The prevalence of pain re-experiencing in this population and its almost complete absence in nonclinical populations supports an account of memory in PTSD in which sensory properties are especially prominent. This finding raises the possibility that some patients with refractory chronic pain may be experiencing PTSD flashbacks.
